# Symbiotic Aerogel Fibers Made via In-Situ Gelation of Aramid Nanofibers with Polyamidoxime for Uranium Extraction

**DOI:** 10.3390/molecules24091821

**Published:** 2019-05-11

**Authors:** Juan Li, Jin Wang, Wei Wang, Xuetong Zhang

**Affiliations:** 1 School of Nano Technology and Nano Bionics, University of Science and Technology of China, Hefei 230026, China; jli2017@sinano.ac.cn; 2 Suzhou Institute of Nano-Tech and Nano-Bionics, Chinese Academy of Sciences, Suzhou 215123, China; jwang2014@sinano.ac.cn (J.W.); winsist@yeah.net (W.W.); 3 Division of Surgery & Interventional Science, University College London, London, NW3 2PF, UK

**Keywords:** aramid nanofiber, polyamidoxime, aerogel, fiber, uranium extraction

## Abstract

The uranium reserve in seawater is enormous, but its concentration is extremely low and plenty of interfering ions exist; therefore, it is a great challenge to extract uranium from seawater with high efficiency and high selectivity. In this work, a symbiotic aerogel fiber (i.e., PAO@ANF) based on polyamidoxime (PAO) and aramid nanofiber (ANF) is designed and fabricated via in-situ gelation of ANF with PAO in dimethyl sulfoxide and subsequent freeze-drying of the corresponding fibrous gel precursor. The resulting flexible porous aerogel fiber possesses high specific surface area (up to 165 m^2^·g^−1^), excellent hydrophilicity and high tensile strength (up to 4.56 MPa) as determined by BET, contact angle, and stress-strain measurements. The batch adsorption experiments indicate that the PAO@ANF aerogel fibers possess a maximal adsorption capacity of uranium up to 262.5 mg·g^−1^, and the absorption process is better fitted by the pseudo-second-order kinetics model and Langmuir isotherm model, indicating an adsorption mechanism of the monolayer chemical adsorption. Moreover, the PAO@ANF aerogel fibers exhibit selective adsorption to uranium in the presence of coexisting ions, and they could well maintain good adsorption ability and integrated porous architecture after five cycles of adsorption–desorption process. It would be expected that the symbiotic aerogel fiber could be produced on a large scale and would find promising application in uranium ion extraction from seawater.

## 1. Introduction

With the increasing demand for eco-friendly energy, nuclear energy has taken on greater importance as it has the characteristic advantages of higher calorific value, lower greenhouse gas emission [1,2,3] and safer operating technique in comparison with the traditional fossil energy [4,5]. Since uranium is the main source in the nuclear industry, efficient and sustainable uranium extraction has attracted increasing interest worldwide [6,7,8,9]. The ocean contains about 4.5 billion tons of uranium, which is nearly 1000 times that of terrestrial uranium reserves and could supply nuclear energy for 13,000 years [10,11,12]. Among various approaches applied to extract uranium from seawater, adsorption is a widely studied process in terms of its high efficiency [13], ease of handling [14] and little pollution to the environment [15,16]. On account of the high chelating affinity of amidoxime groups towards uranyl ions and favorable acid-base resistance, amidoxime-based adsorbents (particularly in the form of polymers), were regarded as one of the most promising candidates for uranium extraction [17,18,19,20,21]. Recently, by means of a half-wave rectified alternating current electrochemical method, Cui et al. developed an approach to extract uranium from seawater with high uranium uptake up to 1932 mg·g^−1^ [22]. Wang et al. reported a mass production of the poly (imide dioxime) nanofiber adsorbent with excellent adsorption capacity of 951 mg·g^−1^ in uranium spiked natural seawater (8 ppm) [21]. However, these methods require some additional electrochemical equipment or air pumps, which will increase the cost of the preparation and use of the adsorbent materials to a certain extent.

The amidoxime-based materials were normally obtained from the chemical modification of nitrile-containing polymers. However, the amidoximated polyacrylonitrile (AO-PAN) could not be directly used as adsorbent owing to their fragility and brittleness. For the purpose of enhancing the mechanical property of the adsorbent, a variety of methods had been adopted by grafting acrylonitrile monomers to strong polymer materials by means of atom-transfer radical polymerization (ATRP) [6] and radiation-induced graft polymerization (RIGP) strategies [17] followed by the post-amidoximation. Those routes require post-polymerization-modification treatment, which is inefficient. However, due to the immiscibility of PAO with many other polymers, it is hard to obtain amidoxime-based composite materials assisted by solution. 

Besides, with regard to the practical application of various adsorbents in seawater, more active chelating sites, more feasible materials synthesis along with less cost are critical factors for the design of the optimal adsorbent towards high efficiency [23,24,25,26]. From this point, aerogels as highly porous materials with extremely high specific surface area (SSA) (~1000 m^2^·g^−1^) and high porosities (~99%) satisfy the above requirements exactly [27,28,29,30,31]. Thus, if the PAO was fabricated into aerogel, it would become a strong absorbent for uranium adsorption due to its large SSA and numerous amidoxime groups. However, synthesis for pure PAO aerogel has not been realized so far. 

**Scheme 1:**
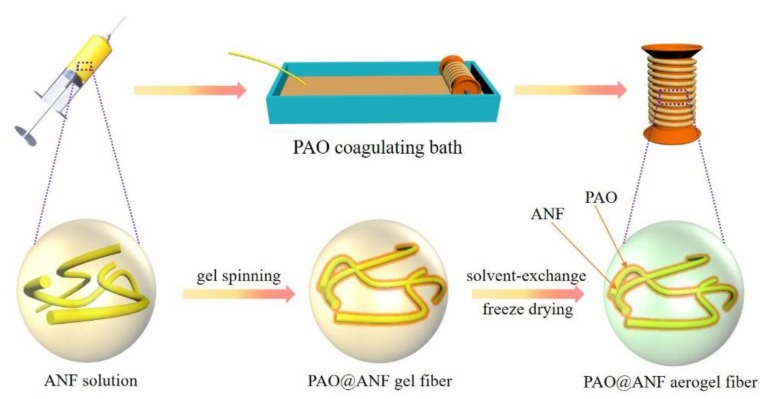
Schematic diagram for the wet spinning of the polyamidoxime aramid nanofiber (PAO@ANF) aerogel fibers.

In this work, we found that physical gel could be formed by mixing PAO with aramid nanofiber (ANF) in dimethyl sulfoxide (DMSO) possibly due to the incipient-network conformal-growth [32] of ANF and PAO via hydrogen bonding, while none of them could be gelled alone in DMSO. Such behavior intrigues us, and has inspired us to design and fabricate a symbiotic aerogel fiber, as illustrated in Scheme 1; ANF was used as both the framework building block [33] and spinning component in DMSO, while DMSO solution containing PAO was used as the coagulating bath. In short, there are three major points in this work to prepare amidoxime-based fiber adsorbent for uranium extraction. First, we developed a facile and efficient method to prepare amidoxime functionalized aerogel fibers (PAO@ANF) by wet-spinning. After the amidoximation of PAN to obtain PAO solution, the PAO@ANF wet-gel fibers were in-situ formed after spinning ANF into PAO, and the resulting PAO@ANF symbiotic aerogel fibers were obtained by freeze-drying of the corresponding gel precursors. Compared to aerogel monoliths, the aerogel fibers could facilitate water diffusion for faster absorption, while compared to aerogel powders or granules, the aerogel fibers could be woven into various textiles for easy collection after use. Interestingly, the PAO@ANF symbiotic aerogel fibers exhibit excellent hydrophilicity, high flexibility, outstanding mechanical strength, large SSA and promising application in uranium adsorption from seawater with the capacity up to 262 mg·g^−1^. Second, this wet-spinning method to fabricate PAO@ANF aerogel fibers is capable of being produced on a large scale. Proved by a simple experiment, the preparation process of PAO@ANF gel fiber is basically continuous without a high DC voltage source of electrospinning method [20,34] or air pump of blow spinning method [12,21]. Furthermore, the preparation rate of PAO@ANF gel fiber could be sped up by setting multiple syringes simply. Last but not least, the PAO@ANF aerogel fibers possess excellent structural stability, except for high adsorption capacity and selectivity towards uranium, which still remains integrated 3D porous structure after adsorption–desorption cycles, indicting excellent resistance to acid corrosion and external mechanical damage. To sum up, this robust, porous and hydrophilic PAO@ANF aerogel fibers, with facile and large-scale preparation, has promising application prospect for uranium extraction from seawater.

## 2. Results and Discussion

### 2.1. Synthesis and Characterization of Fibers

**Figure 1:**
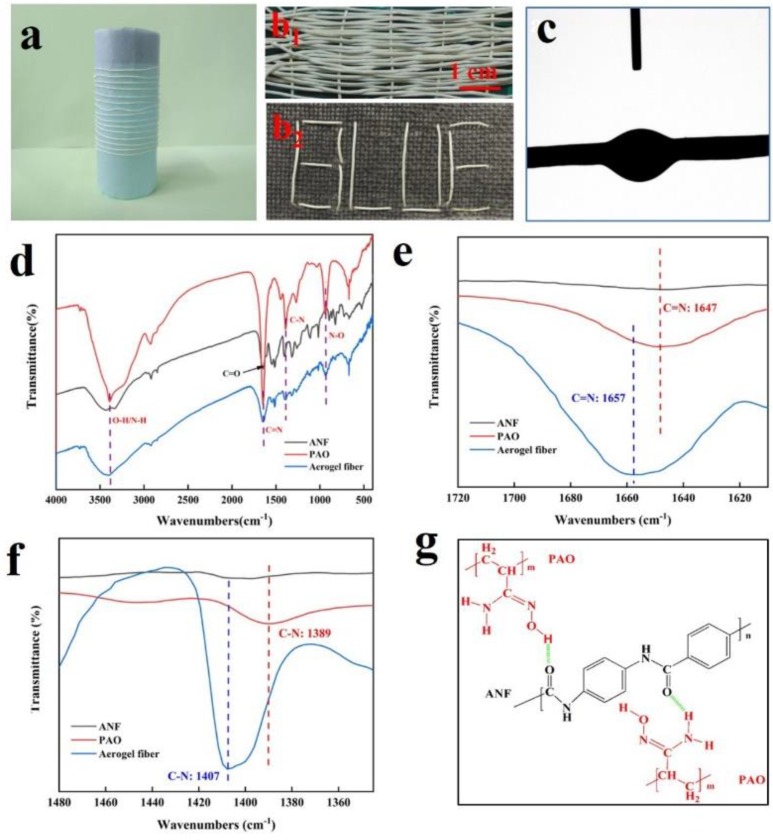
(**a**) Photograph of the PAO@ANF aerogel fibers collected with a roller; (**b_1_**) The free-standing fabric woven from the aerogel fibers; (**b_2_**) a letter pattern knitted on a cotton fabric; (**c**) contact angle of a single PAO@ANF aerogel fiber; (**d**) FTIR spectra of ANF, PAO and PAO@ANF aerogel fiber; and (**e**,**f**) magnified plot of FTIR spectra showing the peak positions associated with the C=N and C-N; (**g**) chemical structure of PAO and ANF, as well as the diagram of their hydrogen bonding interactions.

ANFs are formed by dispersing commercially available Kevlar fibers in basic DMSO solution [33], and they could be transformed into hydrogel simply by replacing DMSO with water [35]. Interestingly, in this work we observed that physical gel was immediately formed after mixing ANF with PAO in DMSO solution, and the continuous spinning of PAO@ANF gel fibers was fulfilled by spinning ANF into a PAO coagulating bath (Figure S1 and Video S1, S2, see the Supplementary Materials). The gel fibers could be freeze-dried to produce continuous aerogel fibers (Figure S1c). Besides, the composition of PAO vs. ANF is controlled by fixing the concentration of PAO at 0.1 g·mL^−1^ while altering the concentration of ANF from 1.0 to 2.0 wt.%. Since neither ANF nor PAO could be gelled alone in DMSO [33,34], the resulted composite aerogel fibers were named as symbiotic ones because the aerogel network was formed by the PAO-ANF composite (which will be confirmed by the SEM images in the following section). PAO macromolecules were attached onto the ANFs via hydrogen bonding and the growth of the ANF in diameter was witnessed by SEM. In the symbiotic aerogel fibers, the ANF served as the critical matrix while the PAO served as the cross-linker as well as the functional unit for uranium absorption. As shown in Figure 1a, the PAO@ANF aerogel fibers are light-yellow, flexible and robust, they could be easily woven into free-standing textile or knitted into a letter pattern on a fabric (Figure 1b_1_,b_2_). The knittable capacity of the aerogel fibers would facilitate the practical uranium adsorption application. The contact angle of the PAO@ANF aerogel fibers was ranged from 24° to 29° (Figure 1c), indicating excellent hydrophilicity of the aerogel fibers, which benefits the uranium adsorption from seawater. 

The amidoximation of PAN and the chemical structure of the PAO@ANF aerogel fibers can be determined by FTIR. As shown in Figure 1d, the FTIR spectrum of the amidoximed PAN (PAO) displays typical bond of amidoxime group at 1647 cm^−1^ (C=N), 1390 cm^−1^ (C-N) and 935 cm^−1^ (N-O) without the appearance of C≡N peak (2246 cm^−1^) [36,37], indicating successful conversion of nitrile groups into the amidoxime groups. Moreover, the FTIR spectrum (Figure 1e,f) and Roman scattering spectroscopy (Figure S2) provide insights into gelation mechanism of PAO@ANF. Two distinct blue-shifts of the peaks corresponding to C=N and C-N in the aerogel fiber (PAO@ANF) could be clearly identified as compared to bare PAO (Figure 1e,f), demonstrate the presence of hydrogen bonds between PAO and ANF (Figure 1g). We speculated that the vital role of hydrogen bond in PAO@ANF composites results in the formation of PAO@ANF gel fiber, which is composed of the aramid nanofiber as a skeleton and PAO coating on the surface of ANF.

**Figure 2:**
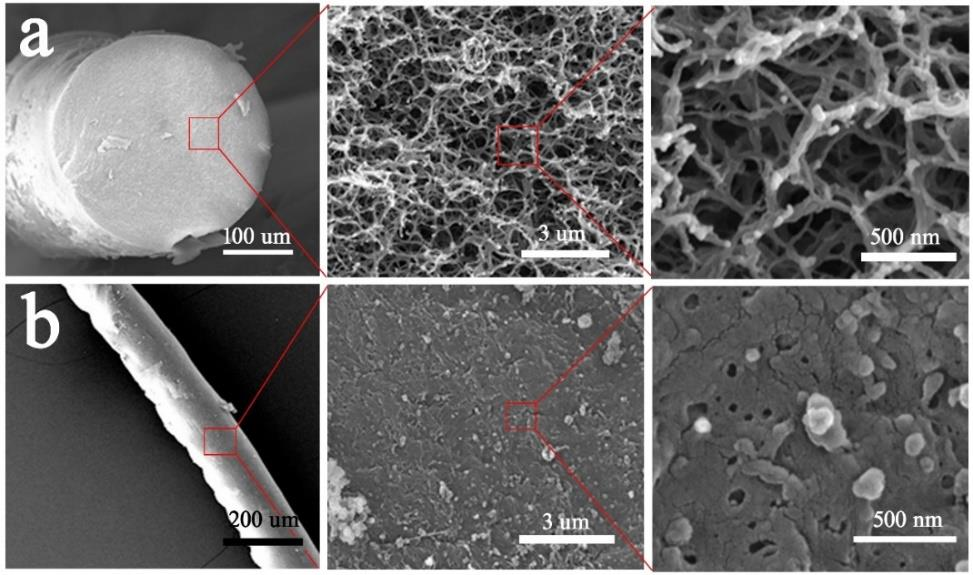
SEM images of (**a**) the cross-section and (**b**) the surface of the PAO@ANF symbiotic aerogel fibers with different magnification.

The microstructure of the aerogel fibers was revealed by SEM. As seen from Figure 2 and Figure S3 in the Supplementary Materials, the PAO@ANF aerogel fibers possess an interconnected porous structure with macropores larger than 100 nm and the network was formed by nanofibers with average diameter from 27–70 nm, which are similar to but thicker than that of pure ANF (ca. 20 nm). The results indicated that the PAO macromolecules were coated on the ANF and the thickness of the PAO layer was deduced to be larger than 7 nm. The surface of aerogel fibers, on the other hand, forms a skin structure with less pores due to the rapid phase separation upon the contact ANF with PAO during the spinning process. Such a skin-core structure may contribute to the excellent mechanical performance of the aerogel fibers. 

**Figure 3:**
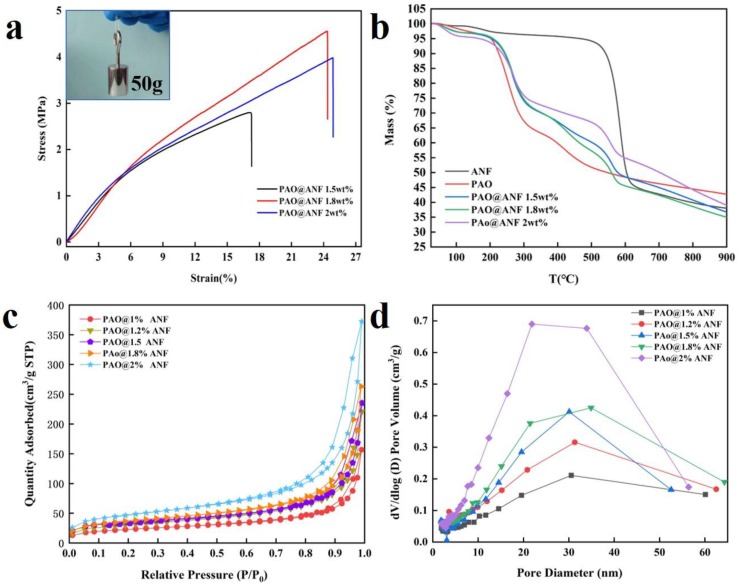
(**a**) The stress–strain curves of the PAO@ANF aerogel fibers formed in ANF solution with concentration from 1.5–2.0 wt.%, inset in e is the photo image of the PAO@ANF aerogel fiber bearing a 50 g weight. (**b**) TGA curves of ANF, PAO, and PAO@ANF aerogel fibers. (**c**) N_2_ adsorption–desorption isotherms and (**d**) pore size distribution curves of the PAO@ANF aerogel fibers.

Figure 3a shows stress–strain curves of the PAO@ANF symbiotic aerogel fibers formed by different concentration of ANF solution from 1.5–2.0 wt.%. The tensile measurements were performed in room temperature. The specimens, in the form of aerogel fiber, were subjected to a tensile extension rate of 1 mm/min. The average diameters of PAO@ANF 1.5–2 wt.% were 242 μm, 235 μm, 238 μm, respectively [38,39]. The average values of elongation and tensile strength at break are 17.36 ± 0.12%, 24.35 ± 0.15%, 24.87 ± 0.12% and 2.81 ± 0.14 MPa, 4.56 ± 0.23 MPa, 4.01 ± 0.16 MPa for the PAO@ANF-1.5 wt.%, PAO@ANF-1.8 wt.%, PAO@ANF-2.0 wt.% aerogel fibers, respectively. The elongation at break of the symbiotic aerogel fibers is much higher than that of the graphene aerogel fibers (4.6–6.2%), while the tensile strength is also higher than or comparable to that of the graphene aerogel fibers (1.45–11.1 MPa) [38,40]. The inset in Figure 3a shows that a single PAO@ANF aerogel fiber can hold a 50 g weight steadily without breaking. We speculated that the mechanical performance of the PAO@ANF aerogel fibers should result from the synergistic effect between ANF and PAO, where the ANF served as the porous matrix for the aerogel fibers. Therefore, the higher the content of ANF, the larger the elongation at break. The excellent mechanical performance of the aerogel fibers benefits these fibers to be used in the natural environment. 

The content of the amidoxime groups could not be determined from the FTIR spectra. Therefore, TGA analysis was carried out as shown in Figure 3b, as well as the DTG curves in Figure S4. The PAO@ANF aerogel fibers exhibited a multi-step decomposition process. The first weight loss before 160 °C was ascribed to the evaporation of physically absorbed water. The second weight loss from 160 to 350 °C could be the decomposition of the amidoxime groups [26,41,42]. The third decomposition from 350–500 °C could be regarded to the decomposition of the main chain of PAO. The last decomposition started from 500 °C could be ascribed to the decomposition of ANF [43,44]. Thus, the content of amidoxime groups could be calculated based on the weight loss between 160 and 350 °C, which is ranged from 22.6 to 26.5 wt.% in the present work. The high content of the amidoxime groups in the aerogel fibers probably indicate a desirable uranium adsorption capacity. Besides, the content of PAO in the symbiotic aerogel fibers also could be roughly calculated, based on the weight loss between 160 and 500 °C (corresponding to the amidoxime groups and the backbone of PAO), to be 28.3, 39.5, and 36.4 wt.% in the PAO@ANF-2.0 wt.%, 1.8 wt.%, and 1.5 wt.% aerogel fibers, respectively. These results indicate that the composition of PAO in the aerogel fibers could be controlled by varying the concentration of ANF in DMSO during the gel spinning step.

Figure 3c,d exhibit the N_2_ adsorption–desorption isotherms and pore size distribution curves of the PAO@ANF symbiotic aerogel fibers prepared from the ANF solution with different concentration, respectively. The detailed information on surface area, pore volume and average diameter has been summarized in Table S1. All the aerogel fibers show the typical “type-IV” isotherm curve with a type H3 hysteresis loop that verifies characteristic mesoporous structure. More importantly, the BET SSA of the aerogel fibers are ranged from 79.6 m^2^·g^−1^ to 164.6 m^2^·g^−1^, and the average pore diameter ranged from 12.2 nm to 14.0 nm. The SSA of the aerogel fibers are higher than that of some other type of polymeric aerogels [31,32] or porous uranium absorbent materials [45,46]. The high SSA of the aerogel fibers is ideal for the uranium absorption from seawater, while the porous structure provides numerous channels for the continuous transportation of water. Therefore, the absorption behavior of the aerogel fibers to uranium is systematically investigated in this work.

### 2.2. Effect of Contact Time and Adsorption Kinetics

**Figure 4:**
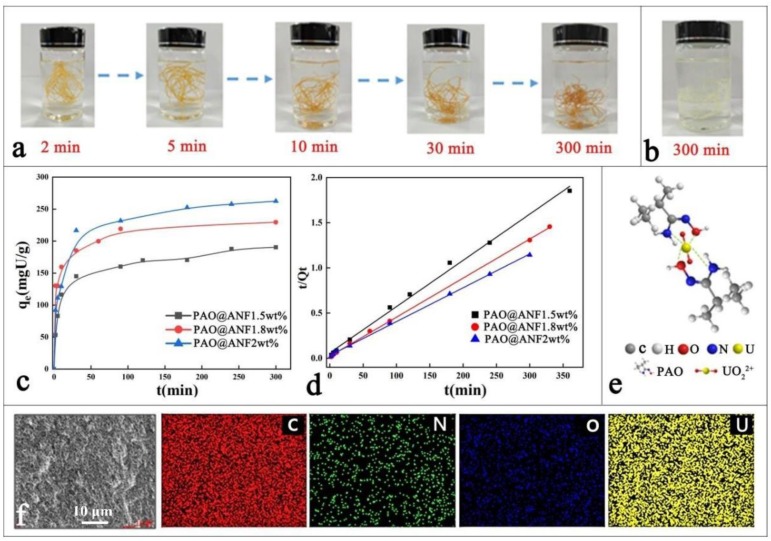
(**a**) Photo images of the PAO@ANF aerogel fibers immersed in uranium solution (1 mg·ml*^−^*^1^) with the time as indicated; (**b**) Photo image of the PAO@ANF aerogel fibers immersed in the deionized water for 5 h; (**c**) Time dependent adsorption of uranium (1 mg·ml*^−^*^1^) by the aerogel fibers (0.5 mg·ml*^−^*^1^); (**d**) Pseudo-second-order kinetic model fits for the adsorption of uranium (1 mg·ml*^−^*^1^) by the aerogel fibers (0.5 mg·ml*^−^*^1^); (**e**) The bonding mechanism between UO_2_^2+^ ion and amidoxime group of PAO@ANF aerogel fiber; (**f**) EDX mapping of various elements in the SEM image of the PAO@ANF aerogel fiber after adsorption of uranium.

A series of experiments on the uranium absorption via the aerogel fibers was designed to study the adsorption capacity and adsorption kinetics. The uranium concentration was determined by V660 spectrophotometer and the calibration curve was shown in Figure S5 in the supporting information. As seen from Figure 4a, the uranium uptake process could be visually observed from the color change of the adsorbent, where the light-yellow aerogel fibers became deeper and turned into brown in 300 min. Such a color change has also been observed in other studies [21]. In comparison, there was no visible change for the aerogel fibers immersed in deionized water for 300 min (Figure 4b). The equilibrium adsorption capacities (q_e_) were presented in Figure 4c, and the adsorption could be divided into two processes: a fast adsorption at the first 10 min, and followed by a slow uptake period until an equilibrium sorption was reached. The highest q_e_ of the aerogel fibers for the uranium is 262.5 mg·g*^−^*^1^, much higher than that of the amidoxime-grafted activated carbon fibers (191.6 mg·g*^−^*^1^) [26], the amidoxime-grated multiwalled carbon nanotubes (206 mg·g*^−^*^1^) [47], and the covalent organic framework (COF) decorated platforms (127 mg·g*^−^*^1^) [48], possibly due to its high SSA. Reducing ANF concentration during spinning results in lower q_e_ value during adsorption, for instance, the q_e_ of the PAO@ANF-1.8 wt% and PAO@ANF-1.5 wt% was 226.7 mg/g and 194.4 mg·g*^−^*^1^, respectively. This trend is coherent with the SSAs and the results suggest that improving the BET surface area of the aerogel fibers may significantly enhance the absorption capacity of the aerogel fibers [30].

To determine the mechanism of the adsorption process, the pseudo-first order and pseudo-second-order kinetics models were employed respectively using the equations below:

(1) $$\ln\left(q_{e}-q_{t}\right){=\mathrm{lnq}}_{e}-k_{1}t$$

(2) $$\frac{t}{q_{t}}=\frac{1}{K_{2}q_{e}^{2}}+\frac{t}{q_{e}}$$

where the q_t_ and q_e_ (mg·g^−1^) refer to the amounts of the uranium adsorbed at time t (min) and at equilibrium, respectively. k_1_ (min^−1^) and k_2_ (g·mg^−1^·min^−1^) are the kinetic constants of the pseudo-first-order and pseudo-second-order models, respectively. As presented in Figure 4d, the uranium absorption exhibited a pseudo-second-order (R^2^ > 0.99) and the highest rate constant of 8.42 × 10^−4^ g·mg^−1^·min^−1^ was achieved for the PAO@ANF-1.8 wt.% aerogel fibers (See Table 1 for the detailed information), which is much higher than that of hydrogel (8.5 × 10^−5^ g·mg^−1^·min^−1^) [49] and microsphere (1.3 × 10^−4^ g·mg^−1^·min^−1^) [50] reported elsewhere. The plotted curves and parameters of the pseudo-first-order models were also shown in Figure S6 and Table S2. Moreover, the theoretically calculated equilibrium adsorption capacity (q_e,cal_) is nearly the same as the measured one (q_e, exp_). The result indicated that chemical adsorption is the dominating factor. In fact, uranium has hardly been absorbed by physical absorption, as depicted in Figure 4e, the uranium ions always form complexes with amidoxime groups in the form of UO_2_^2+^, thus show high selectivity. Figure 4f presents the cross section EDX spectra of the PAO@ANF aerogel fiber. The existence of C, N, O, and U can be clearly observed, and all the elements are homogeneously distributed in the aerogel. This also indicates that the PAO are well dispersed in the matrix.

### 2.3. Equilibrium Adsorption

**Figure 5:**
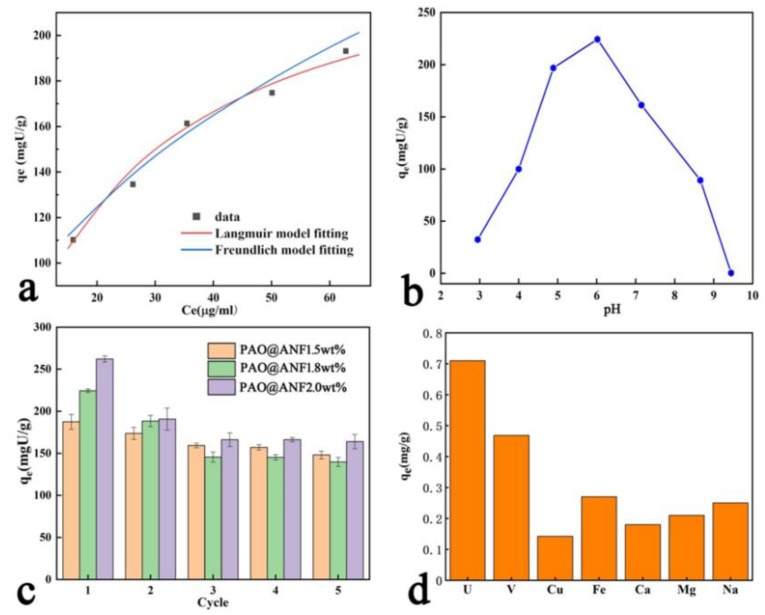
(**a**) Equilibrium adsorption isotherms fitted with Langmuir model and Freundlich model; (**b**) the effect of initial pH value on the adsorption capacity of the PAO@ ANF(2 wt.%) aerogel fiber; (**c**) Uranium adsorption performance in five adsorption–desorption cycles by using PAO@ANF aerogel fiber; (**d**) Effect of coexisting metal ions on the U(VI) adsorption capability.

In order to further clarify the adsorption mechanism, the adsorption isotherm experiment with different initial concentration of uranium (20–180 μg·mL^−1^) was conducted at 298 K. The equilibrium adsorption isotherms were exhibited in Figure 5a. Both Langmuir model and Freundlich model were applied to analyze the thermodynamic behavior. The equations are below:

(3) $$\frac{C_{e}}{q_{e}}=\frac{1}{bq_{m}}+\frac{C_{e}}{q_{m}}$$

(4) $$\ln q_{e}=\ln k_{F}+\frac{1}{n}C_{e}$$

where q_m_ (mg·g^−1^) and b are the maximum adsorption capacity and the Langmuir constant (mL·μg^−1^), respectively, q_e_ is the uranium adsorbed amount at the equilibrium, n is an empirical parameter related to the intensity of sorption, C_e_ is the equilibrium concentration (μg·mL^−1^), k_F_ refers to the Freundlich constant (mg/g) × (μg/mL)^1 − 1/n^ related to the sorption capacity of the adsorbent. The results are shown in Table 2. Regarding the correlation coefficient R^2^, the adsorption process was well described by the Langmuir model (R^2^ = 0.9967), indicating that the monolayer chemical adsorption was the dominant form. The result further indicated that the uranium may be selectively absorbed by the amidoxime group. Moreover, the calculated value of equilibrium uptake capacity (252 mg·g^−1^) was nearly equivalent to the experimental value (267 mg·g^−1^).

### 2.4. Effect of PH

In order to further clarify the adsorption mechanism, the adsorption isotherm experiment with different initial concentration of uranium (20–180 μg·mL^−1^) was conducted at 298 K. The equilibrium adsorption isotherms were exhibited in Figure 5a. Both Langmuir model and Freundlich model were applied to analyze the thermodynamic behavior. The equations are below:

Importantly, the pH value of the solution may affect the adsorption performance of the aerogel fibers. A series of experiments was carried out by adding 10 mg PAO@ANF aerogel fibers in 25 mL uranium solution (100 ppm) with pH values ranged in 3.0–10.0. As shown in Figure 5b, the uranium adsorption capacity of the aerogel fibers increases with the increase of pH value from 3.0–6.0 and then decreases with the increase of pH value from 6.0–10.0. We notice that, at the point of pH 6.0, the adsorbent reaches a maximum amount of uranium uptake. According to previous reports [51,52], under acidic condition, the low adsorption capacities of PAO@ANF aerogel fibers mainly result from electrostatic repulsion between uranium in the primary form of UO_2_^2+^ and positively charged protonated adsorbent surface. With the augment of pH, the adsorbent gradually deprotonated and exhibited increased uranium adsorption capacity. When the solution pH is 6.0, then adsorbent reaches the best adsorption performance because of the synergistic effect of electrostatic interaction and complexation of uranyl ions with amidoxime groups. However, in an alkaline environment, U (VI) exists in the partial form of negatively charged hydration ions, such as (UO_2_)^3^(OH)_8_^2−^, (UO_2_)_3_(OH)_10_^4−^ and UO_2_(OH)_3_^−^ etc., and it is difficult for them to interact with amidoxime groups, leading to the decline of adsorption efficiency. 

### 2.5. Reusability 

Reusability is an important evaluation criterion for determining whether the adsorbent could be put into real application or not. In this work, for the study of the regeneration of the PAO@ANF symbiotic aerogel fibers, hydrochloric acid solution (0.7 M) was used as an eluent to remove uranium ions from the adsorbent after adsorption. As shown in Figure 5c, all three kinds of aerogel fibers can keep more than 65% adsorption capacity after five cycles, and the adsorption capacity became stable after three cycles. The decline in the adsorption performance may result from the structure damage during repeated adsorption–desorption-freeze drying cycles and incomplete removal of uranium on the adsorbent. The morphology of the aerogel fibers after five-recycle use was investigated by SEM. As depicted in Figure S7 in the Supporting Information, the microporous framework of the PAO@ANF aerogel fibers was well retained, which provides a possibility for larger active area to combine uranium ions after adsorbent regeneration. Consequently, the reusability and structural stability mean that the PAO@ANF aerogel fibers are great potential candidates for uranium extraction from seawater.

### 2.6. Selective Adsorption in Simulated Seawater

Selectivity plays another important role in uranium adsorption performance from seawater because there are plenty of different kinds of metal ions in seawater. Therefore, the competitive adsorption of the uranium ions in the presence of various ions, such as VO^3+^, Cu^2+^, Fe^3+^, Ca^2+^, Mg^2+^, Na^+^, with the PAO@ANF aerogel fibers was investigated, the initial concentration of the uranyl ions and other interfering ions were listed in Table S3 in the Supporting Information. The concentration of the ions is similar to that of seawater. The results were given in Figure 5d. Obviously, in the presence of various competitive ions, the adsorbent possesses the highest adsorption capacity for uranium, reaching more than 0.7 mg·g^−1^, and shows a higher absorption capacity over VO^3+^(0.47 mg·g^−1^), which is comparable to the literatures reported elsewhere [12,21,53]. By analyzing the above results, we can draw a conclusion that the PAO@ANF aerogel fiber adsorbents have the super selectivity for uranium, due to the strong complexation of amidoxime groups in the PAO@ANF aerogel fibers with UO_2_^2+^.

## 3. Materials and Methods 

### 3.1. Materials

Kevlar 1000D was obtained from Dongguan SOVETL Co., Ltd. (Dongguan, China). Uranyl nitrate was purchased from Hubei Chushengwei Chemistry Co., Ltd. (Wuhan, China). Polyacrylonitrile (PAN, AR, m_w_ = 150000, Macklin), hydroxylamine hydrochloride (NH_2_·OH·HCl, AR, Aladdin, Shanghai, China, chlorophosphonazo III (CPA III, AR, Aladdin), tert-butyl alcohol (TBA, AR, Aladdin) were used without further purification. Dimethyl sulfoxide (DMSO, AR, Sinopharm Chemical Reagent Co., Ltd., Shanghai, China), potassium hydroxide (KOH, AR), sodium hydroxide (NaOH, AR), hydrochloric acid (HCl, AR) were all purchased from Sinopharm Chemical Reagent Co., Ltd. 1.0–2.0 wt.% ANF dispersions in DMSO were prepared in our own lab according to that which is recorded in the literature [33]. The water used in the experiments was deionized. All the other agents were used as received.

### 3.2. Preparation of the PAO Coagulating Bath 

The amidoximation of PAN: The experimental procedure was referenced from the previously reported literature [34]. NH_2_·OH·HCl (50 g) was completely dissolved in DMSO (150 mL), then NaOH was added into the solution for 0.5 h. Finally, PAN powder (15 g) was added into the solution for another 0.5 h. In this process, mixing as well as subsequent dissolving is along with vigorous mechanical stirring at room temperature. After that, the mixture was kept at 75 °C for 7 h, then cooled to room temperature and filtered to obtain the filtrate for spinning directly. The concentration of PAO in the filtrate is about 0.1 g·mL^−1^.

### 3.3. Preparation of PAO@ANF Symbiotic Aerogel Fibers

As illustrated in Scheme 1, the ANF solution (1.0–2.0 wt%) was spun into the PAO coagulating bath. This was followed by washing the gel fibers at least five times with water to replace the DMSO containing KOH. Then the hydrogel fibers were solvent-exchanged with the mixture of water/TBA (mass ratio 4:1) for at least 4 times. Finally, the PAO@ANF aerogel fibers were obtained by freeze-drying of the corresponding gel precursors. 

### 3.4. Characterization

The morphology of samples was performed by scanning electron microscopy (SEM) (FEI Quanta 400 FEG, Hillsboro, OR, USA), operating with the acceleration voltage of 5–15 kV. Fourier Transform Infrared Spectrometer (FT-IR) was carried out using Thermo Fisher Scientific Nicolet 6700 (Waltham, MA, USA). Raman spectra were recorded on a LabRAM HR Raman spectrometer (Paris, France) with a 50 mW He-Ne laser operating at 632 nm with a CCD detector. The contact angle was measured by OCA 15EC Data Physics Instruments GmbH (Stuttgart, Germany). The stress–strain curves were examined by using an Instron 3365 tensile testing machine. Thermal gravimetric analysis (TGA) curves were recorded on a TG 209F1 Libra (NETZSCH, Bavaria, Germany) analyzer with a heating rate of 10 °C min^−1^ in a nitrogen atmosphere. The SSA of the PAO@ANF aerogel fiber was determined by the Brunauer–Emmett–Teller (BET) method, based on the amount of N_2_ adsorbed at pressures 0.05 < *P*/*P*_0_ < 0.3. The pore size distribution and average pore diameter were analyzed by the BJH nitrogen adsorption and desorption method (ASAP 2020, Micromeritics, Atlanta, GA, USA). 

### 3.5. Batch Adsorption Experiments

Batch adsorption tests were carried out in uranium (VI) solution (mg·mL^−1^ or μg·mL^−1^) to assess the uranium (VI) sorption to PAO@ANF aerogel fibers. A uranium concentration of 1 mg·mL^−1^ was obtained by dissolving 827.7 mg of uranyl nitrate salt (UO_2_(NO_3_)_2_) in 500 mL of DI water. 10 mg adsorbents were stirred in 20 mL uranium (VI) solution for 8 h at 298 K. The concentration of uranium was analyzed using UV-Vis spectrophotometer (Agilent Cary5000, Palo Alto, CA, USA) [23,24]. The uptake capacity of the adsorbent was calculated using equation (5) as follows:

(5) $$q_{e}=\left(C_{0}-C_{e}\right)\times \frac{V}{m}$$

where q_e_ (mg·g^−1^) is the adsorption capacity of the adsorbent reaching the uptake equilibrium, C_0_ and C_e_ (mg·mL^−1^) are the original and the equilibrium concentrations of the uranium ions, V (mL) is the volume of the solution, m (mg) is the weight of the added fibrous adsorbent.

## 4. Conclusions

A series of PAO@ANF symbiotic aerogel fibers was developed by the direct spinning of ANF solution into PAO coagulating bath and followed by freeze-drying. The fibers were robust with an elongation up to 25% and a high tensile strength of 4.56 MPa at break. The diameter and SSA of the aerogel fibers are controllable in the range of 170–500 μm and 80–165 m^2^·g^−1^, respectively. The SEM results indicate that the aerogel fibers are built up with nanofibrous building blocks. Due to the high SSAs and the abundant amidoxime groups, the aerogel fibers exhibit enhanced uptake performance for uranium with the maximal adsorption capacity of 262 mg·g^−1^. Besides, the aerogel fibers could maintain good adsorption ability and integrated porous architecture after five adsorption–desorption cycles, and they show high selective absorption for uranium in the presence of various competing ions. Regarding the short fabrication time and the facile processing, the PAO@ANF symbiotic aerogel fibers could easily be scaled up and serve as an ideal adsorbent to extract uranium ions from seawater.

## Figures and Tables

**Table 1:** The adsorption kinetic fitting parameters of pseudo-second-order kinetic model with the PAO@ANF aerogel fiber adsorbents.

Adsorbents	K (g·mg^−1^·min^−1^)	q_e,exp_ (mg·g^−1^)	q_e,cal_ (mg·g^−1^)	R^2^
PAO@ANF 1.5wt%	4.39 × 10^−4^	194.36	195.31	0.9953
PAO@ANF 1.8wt%	8.42 × 10^−4^	226.74	231.48	0.9995
PAO@ANF 2.0wt%	4.50 × 10^−4^	262.50	267.38	0.9991

**Table 2:** Langmuir and Freundlich constants and correlation coefficients.

Langmuir			Freundlich		
q_max_ (mg/g)	b (mL/μg)	R^2^	K_F_ ((mg/g) × (μg/mL) ^1 − 1/n^)	n	R^2^
251.89	0.0487	0.9967	37.39	2.505	0.9846

## Supplementary Materials

The following are available online. Figure S1: The photos of a device on wet spinning of the PAO@ANF gel fiber, the PAO@ANF gel fibers; and the PAO@ANF aerogel fibers. Figure S2: Roman scattering spectroscopy of ANF, PAO and PAO@ANF aerogel fiber. Figure S3: SEM images the photo image and the contact angel of the PAO@ANF(1.5wt.%) aerogel fiber and PAO@ANF(1.8wt.%) aerogel fiber. Figure S4: DTG curves of ANF, PAO, and PAO@ANF aerogel fibers. Figure S5: The relation between absorbency and volume of CPA Ⅲ and HCl solution (0.6 M), as well as absorption curve of complex and CPA Ⅲ under different dosage of uranyl ions solution (1mgU/mL), calibration curve of uranium. Figure S6: Pseudo-first-order kinetic model fits for the adsorption of uranium (1 mg·mL^−1^) by the aerogel fibers (0.5 mg·mL^−1^). Figure S7: SEM images of the cross section of PAO@ANF 1.5 wt.% aerogel fibers, PAO@ANF 1.8 wt% aerogel fibers and PAO@ANF 2.0 wt.% aerogel fibers after five adsorption–desorption cycles. Table S1: The pore volume and S_BET_ of PAO@ANF aerogel fibers formed by different concentration of ANF solution from 1 wt.%–2 wt.%. Table S2: The adsorption kinetic fitting parameters of pseudo-first-order kinetic model with PAO@ANF aerogel fiber adsorbents. Table S3: Coexisting metal ions concentration prepared for the analysis of the selective adsorption of uranium by the PAO@ANF aerogel fibers. Video S1: The record of the wet gel spinning process of PAO@ANF gel fiber. Video S2: The record of the wet gel spinning process of PAO@ANF gel fiber on a larger scale to verify the possibility of mass continuous production.
