# Supplementary material for: Symbiotic Aerogel Fibers Made via In-Situ Gelation of Aramid Nanofibers with Polyamidoxime for Uranium Extraction
Source: Molecules. 2019 May 11;24(9):1821. doi: 10.3390/molecules24091821 (PMC6539675; doi:10.3390/molecules24091821)
Supplement: Supplementary file 1 [file molecules-24-01821-s001.zip › supplementary materials/Supplementary Figures and Tables.pdf]

molecules

MDPI

Supplementary Materials

Symbiotic Aerogel Fibers Made via In-situ Gelation of Aramid Nanofibers with Polyamidoxime for Uranium Extraction

Juan Li [1,2,†], Jin Wang[2,†], Wei Wang [2] and Xuetong Zhang [2,3] *

[1] School of Nano Technology and Nano Bionics, University of Science and Technology of China, Hefei 230026, China; jli2017@sinano.ac.cn
[2] Suzhou Institute of Nano-Tech and Nano-Bionics, Chinese Academy of Sciences, Suzhou 215123, China; jwang2014@sinano.ac.cn (J.W.); winsist@yeah.net (W.W.)
[3] Division of Surgery & Interventional Science, University College London, London, NW3 2PF, UK
* Correspondence: xtzhang2013@sinano.ac.cn or xuetong.zhang@ucl.ac.uk; Tel.: +86-51-62872821
† These authors contributed equally to the work.

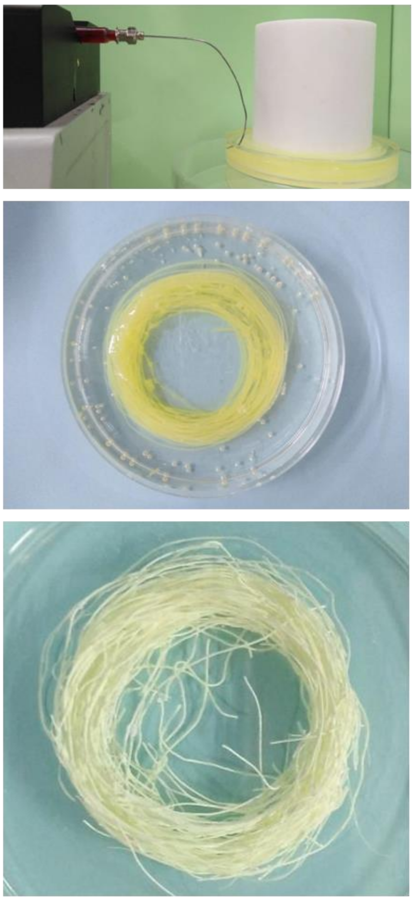

Figure S1. The photos of (**a**) a device on wet spinning of the PAO@ANF gel fiber; (**b**) the PAO@ANF gel fibers; and (**c**) the PAO@ANF aerogel fibers.

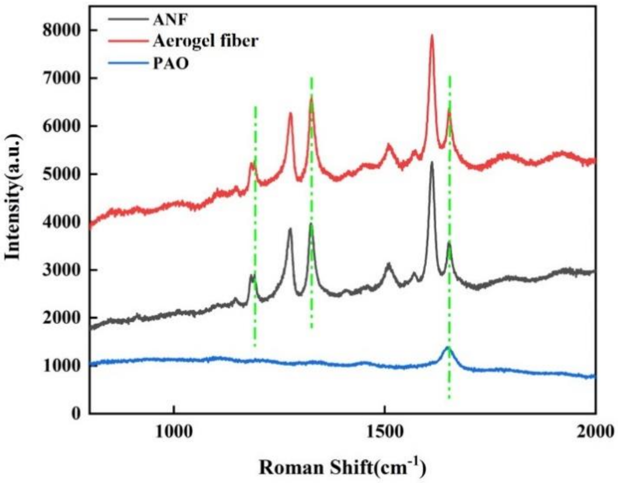

Figure S2. Roman scattering spectroscopy of ANF, PAO and PAO@ANF aerogel fiber.

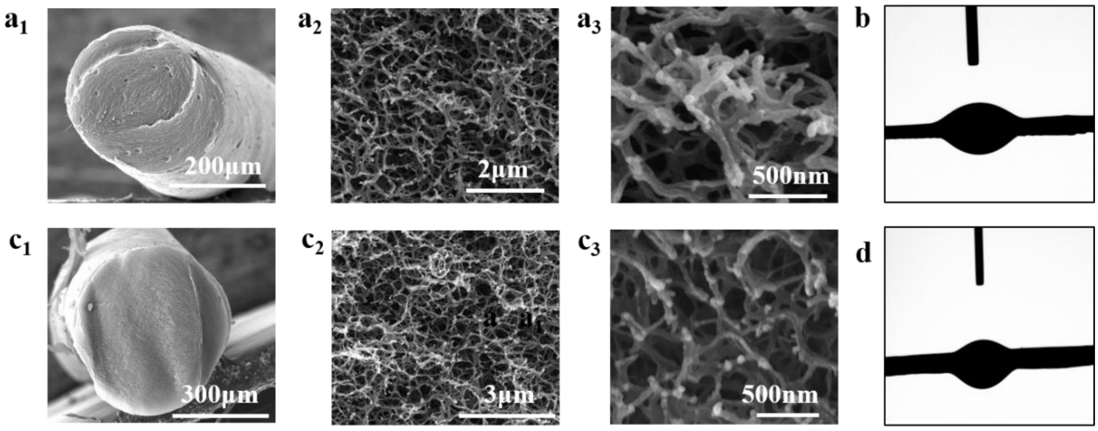

Figure S3. (**a**) SEM images of the PAO@ANF(1.5 wt.%) aerogel fiber at different magnifications; (**b**) the photo image of the contact angel of the PAO@ANF(1.5 wt.%) aerogel fiber; (**c**) SEM images of the PAO@ANF(1.8 wt.%) aerogel fiber at different magnifications; (**d**) the photo image of the contact angel test of the PAO@ANF(1.8 wt.%) aerogel fiber.

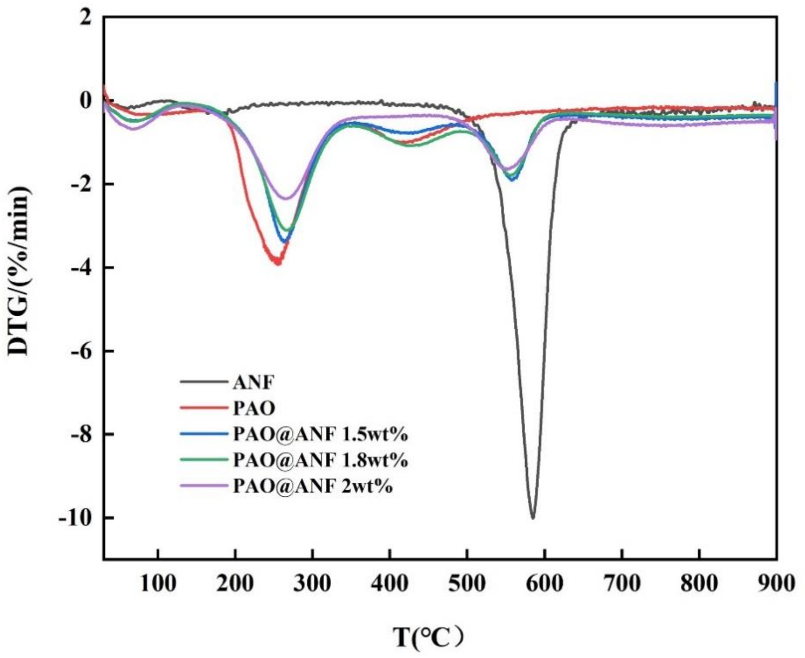

Figure S4. DTG curves of ANF, PAO, and PAO@ANF aerogel fibers. For the calculation of weight content of PAO, the weight percentage of PAO from 160 to 500 °C were adopted. The mass percentage of the aerogel at 160 °C was 96.6, 96.8, 95.3% for PAO@ANF1.5wt%, 1.8wt%, and 2.0wt%, respectively. While the mass percentage of the aerogel at 500 °C was 60.2, 57.3, and 67 % for PAO@ANF 1.5wt.%, 1.8 wt.%, and 2.0 wt.%, respectively. Therefore, the content of PAO in PAO@ANF 1.5wt.%, 1.8 wt.%, and 2.0 wt.% could be calculated to be (96.6−60.2 = 36.4) 36.4, (96.8−57.3 = 39.5) 39.5, and (95.3−67 = 28.3) 28.3wt.%.

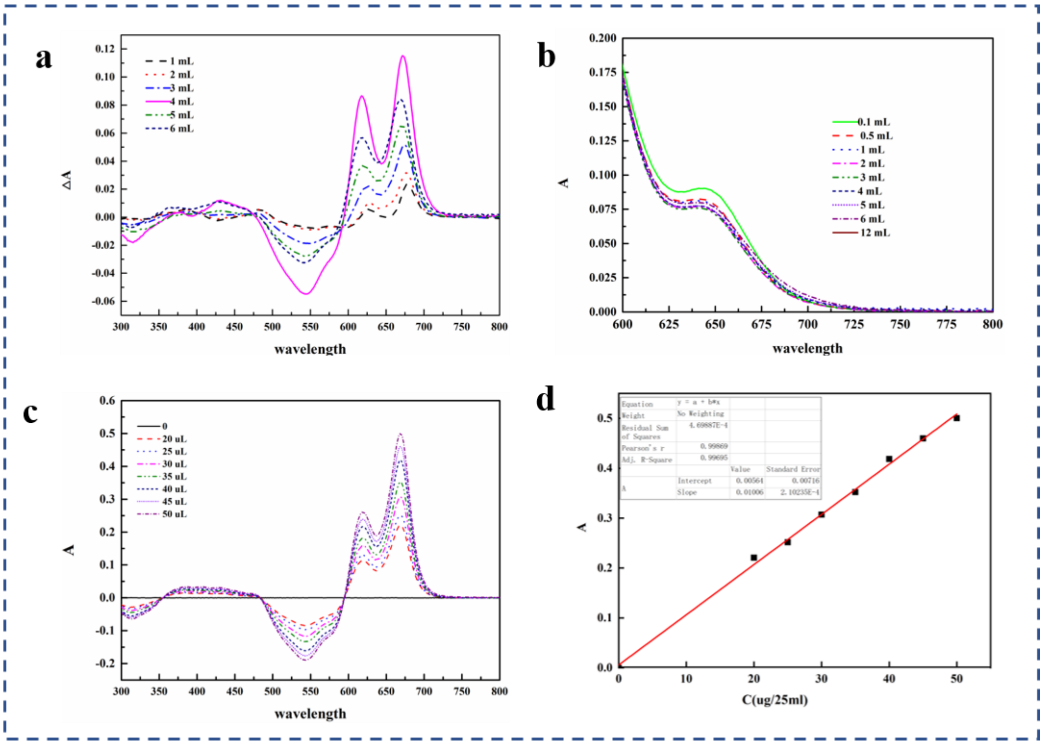

Figure S5. The relation between absorbency and volume of (**a**) CPA Ⅲ and (**b**) HCl solution (0.6 M). (**c**) Absorption curve of complex and CPA Ⅲ under different dosage of uranyl ions solution (1mgU/mL). (**d**) Calibration curve of uranium.

1 mg·mL⁻¹ of uranium concentration was obtained by 0.8277 g of uranyl nitrate salt ($UO_2(NO_3)_2$) dissolved in 500 mL of DI water. Measure accurately 40 μL of uranyl ion solution to be tested and add it to 25 mL volumetric flask. Then, 4 ml CPA Ⅲ solution (0.1mmol/L) and 0.1 ml HCl solution (6 M) was added in sequence followed by adding distilled water up to metered volume of 25ml with shaking until the solution dispersing evenly. After about 15 min, the absorbance A of the solution as prepared was measured by V660 spectrophotometer. The absorbance at the wavelength of 669 nm was taken, and the uranium concentration value was obtained by comparing the calibration curve of uranium.

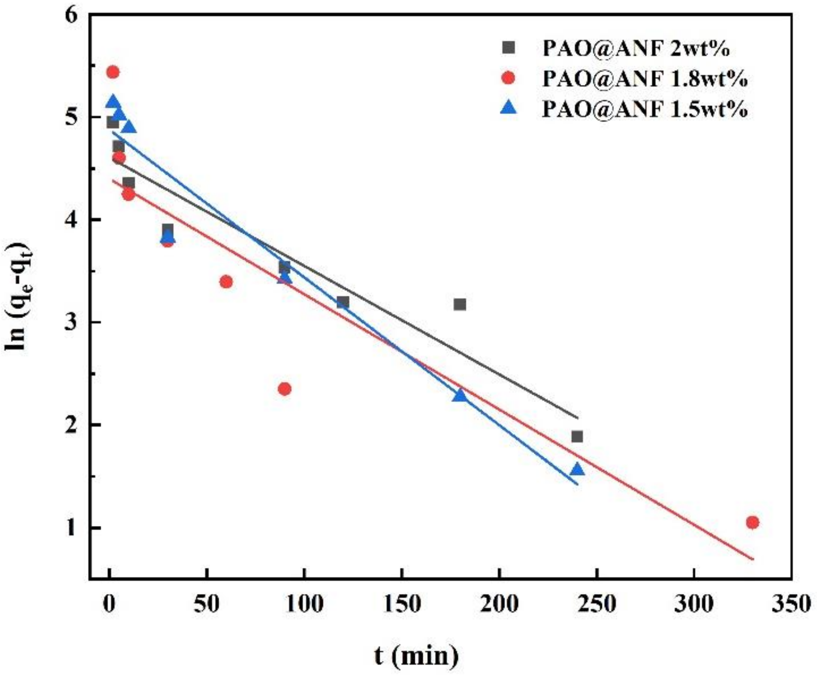

Figure S6. Pseudo-first-order kinetic model fits for the adsorption of uranium (1 mg·mL^{-1}) by the aerogel fibers (0.5 mg·mL^{-1}).

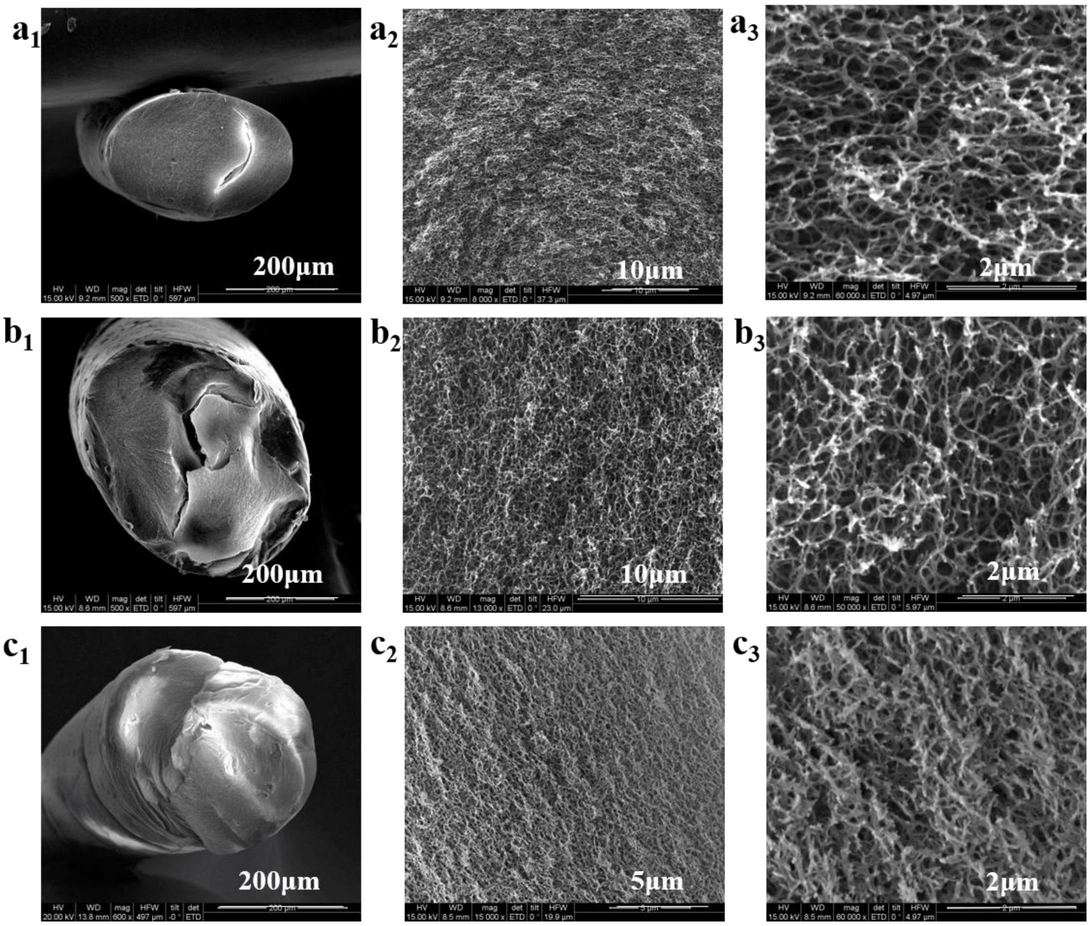

Figure S7. SEM images of the cross section of (**a**) PAO@ANF 1.5 wt.% aerogel fibers; (**b**) PAO@ANF 1.8 wt.% aerogel fibers; (**c**) PAO@ANF 2.0 wt.% aerogel fibers after five adsorption-desorption cycles.

Table S1. The pore volume and S_{BET} of PAO@ANF aerogel fibers formed by different concentration of ANF solution from 1 wt.%–2 wt.%.

Adsorbent	BET Surface Area (m^2/g)	Pore Volume (cm^3/g)	Pore Diameter (nm)
PAO@ANF1.0wt%	79.43	0.24	12.20
PAO@ANF1.2wt%	106.35	0.34	12.87
PAO@ANF1.5wt%	119.67	0.36	12.68
PAO@ANF1.8wt%	128.73	0.40	12.65
PAO@ANF2wt%	164.64	0.58	13.99

Table S2. The adsorption kinetic fitting parameters of pseudo-first-order kinetic model with PAO@ANF aerogel fiber adsorbents.

Adsorbents	K (min^{-1})	$q_{e,exp}$ ($mg \cdot g^{-1}$)	$q_{e,cal}$ ($mg \cdot g^{-1}$)	R^2
PAO@ANF 1.5wt.%	0.0106	194.36	100.24	0.9100
PAO@ANF 1.8wt.%	0.0112	226.74	81.39	0.8005
PAO@ANF 2.0wt.%	0.0144	262.50	131.43	0.9513

Table S3. Coexisting metal ions concentration prepared for the analysis of the selective adsorption of uranium by the PAO@ANF aerogel fibers.

**Element**	**U**	**V**	**Fe**	**Cu**	**Ca**	**Mg**	**Na**
C_0 (ppm)	300×10^{-3}	200×10^{-3}	150×10^{-3}	60×10^{-3}	40	130	109
C_e (ppm)	15.42×10^{-3}	12.74×10^{-3}	42×10^{-3}	3.24×10^{-3}	39.93	129.92	108.9

Video S1. The record of the wet gel spinning process of PAO/ANF fiber.
Video S2. The record of the wet gel spinning process of PAO/ANF fiber on a larger scale to verify the possibility of mass continuous production
